# Exercise-stimulated interleukin-15 is controlled by AMPK and regulates skin metabolism and aging

**DOI:** 10.1111/acel.12341

**Published:** 2015-04-22

**Authors:** Justin D Crane, Lauren G MacNeil, James S Lally, Rebecca J Ford, Adam L Bujak, Ikdip K Brar, Bruce E Kemp, Sandeep Raha, Gregory R Steinberg, Mark A Tarnopolsky

**Affiliations:** 1 Department of Kinesiology, McMaster UniversityHamilton, Ontario, Canada; 2 Department of Pediatrics, McMaster UniversityHamilton, Ontario, Canada; 3 Division of Endocrinology and Metabolism, Department of Medicine, McMaster UniversityHamilton, Ontario, Canada; 4 Department of Medicine, St. Vincent’s Institute of Medical Research, University of MelbourneFitzroy, Vic., Australia; 5 Department of Biochemistry and Biomedical Sciences, McMaster UniversityHamilton, Ontario, Canada

**Keywords:** aging, exercise, muscle, metabolism, mitochondria, skin

## Abstract

Aging is commonly associated with a structural deterioration of skin that compromises its barrier function, healing, and susceptibility to disease. Several lines of evidence show that these changes are driven largely by impaired tissue mitochondrial metabolism. While exercise is associated with numerous health benefits, there is no evidence that it affects skin tissue or that endocrine muscle-to-skin signaling occurs. We demonstrate that endurance exercise attenuates age-associated changes to skin in humans and mice and identify exercise-induced IL-15 as a novel regulator of mitochondrial function in aging skin. We show that exercise controls IL-15 expression in part through skeletal muscle AMP-activated protein kinase (AMPK), a central regulator of metabolism, and that the elimination of muscle AMPK causes a deterioration of skin structure. Finally, we establish that daily IL-15 therapy mimics some of the anti-aging effects of exercise on muscle and skin in mice. Thus, we elucidate a mechanism by which exercise confers health benefits to skin and suggest that low-dose IL-15 therapy may prove to be a beneficial strategy to attenuate skin aging.

## Introduction

Skin is the largest organ in the human body and the primary physical barrier against infection and disease. Aging is associated with the deterioration of the dermal and epidermal layers of the skin, resulting from reductions in cell proliferation, collagen synthesis, extracellular matrix remodeling, and altered epidermal morphology (Fisher *et al*., 2002). These pathophysiological changes are thought to be driven by aged and senescent cells that exhibit reduced energy metabolism, higher mitochondrial oxidative stress, and pronounced mitochondrial DNA (mtDNA) deletions (Isobe *et al*., 1998; Lu *et al*., 1999), reflecting characteristics of the mitochondrial free radical theory of aging (Harman, 1972). In agreement, the deletion of a free radical scavenger within the mitochondria, superoxide dismutase 2, from connective tissue results in premature skin aging (Treiber *et al*., 2011; Velarde *et al*., 2012) and depleting mtDNA in dermal fibroblasts mimics the gene profile of photoaging (Schroeder *et al*., 2008). Conversely, treatment with PPAR agonists that stimulate mitochondrial metabolism and cell proliferation improves skin wound healing (Ham *et al*., 2010) and retards age-related tissue degeneration (Dillon *et al*., 2012). Therefore, interventions that improve skin metabolism and mitochondrial function are a promising means to maintain skin health in old age.

Endurance exercise is commonly prescribed to improve the health and function of the whole body via its ability to improve energy metabolism. Individuals that adhere to a regular exercise program have lower comorbidities and disease risk and exhibit greater survival in later life (Chakravarty *et al*., 2008), but the underlying mediators of these benefits are incompletely understood. Recently, we have shown that exercise training can prevent the systemic mitochondrial dysfunction and progeroid symptoms in the polymerase-gamma mutator mouse, including a prevention of skin deterioration (Safdar *et al*., 2011). Given the systemic nature of the benefits of physical activity, it seems reasonable that circulating factors induced by exercise are likely to mediate at least a portion of these adaptations. Indeed, several proteins secreted from skeletal muscle (myokines) have been identified that can mediate important physiological functions in other organs (Pedersen & Febbraio, 2012), demonstrating a tremendous potential for peripheral tissue metabolic control by exercise-mediated factors.

While facets of skin aging have been ascribed to extracellular hormones that are altered during the lifespan, such as the growth hormone/IGF-1 axis (Zouboulis & Makrantonaki, 2012) or cytokines (Coppé *et al*., 2008), the endocrine effects of exercise on skin or its aging process have not been studied. In the current study, we provide the first knowledge that regular exercise can attenuate skin aging in humans and mice. We show that exercise regulates skin mitochondrial metabolism and that mice lacking muscle AMPK have reduced serum IL-15 and accelerated skin aging. Finally, we partially rescue age-associated skin deterioration to a similar extent as exercise by injecting aged mice with recombinant IL-15, demonstrating the critical role of this factor in skin function and metabolism.

## Results

**Fig 1:**
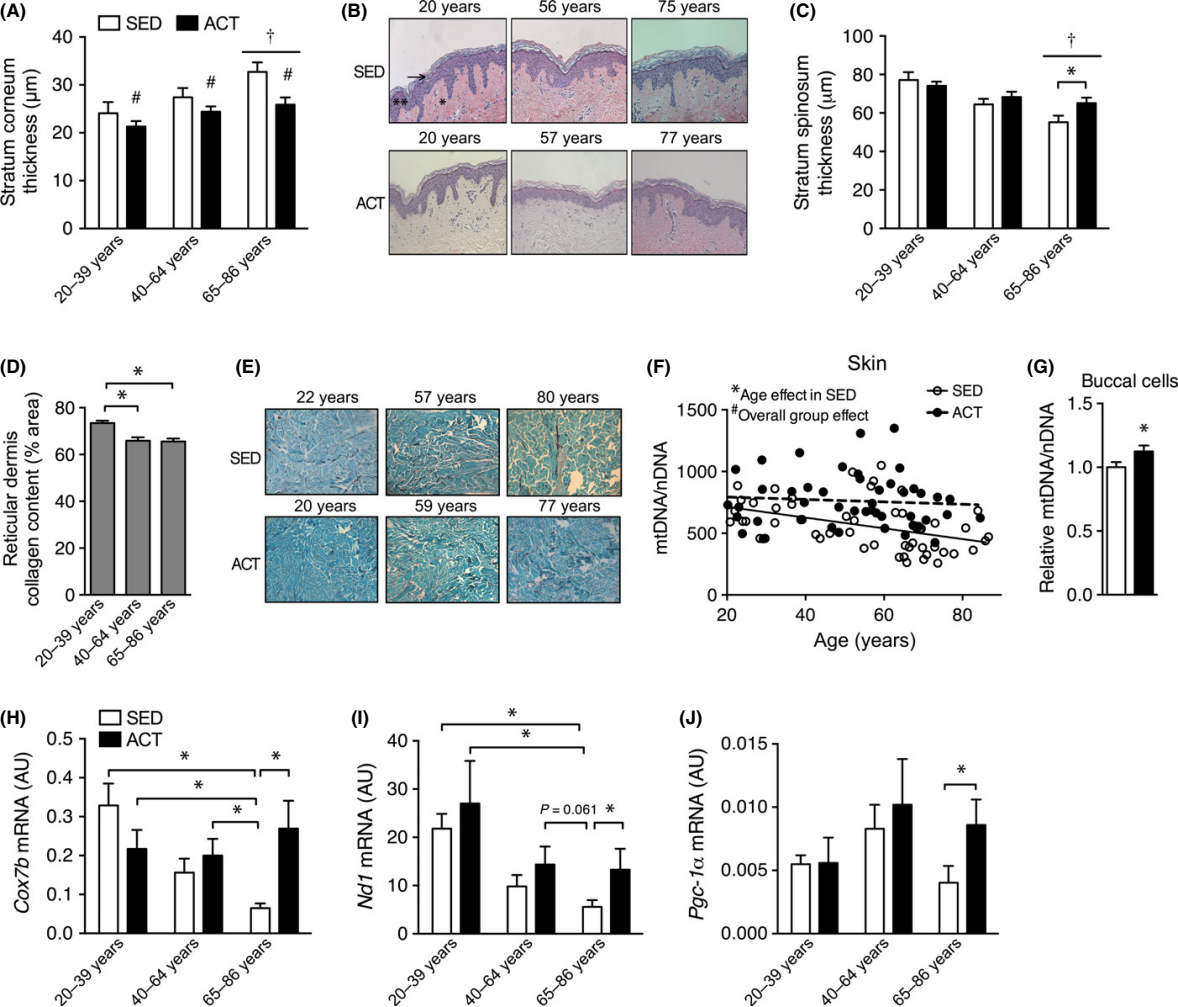
Chronic exercise adherence is associated with attenuated aging in human skin and greater mitochondrial content. (A) Stratum corneum thickness and (B) representative images of skin cross sections stained using H&E from 20- to 86-year old subjects that were highly active exercisers (ACT) or were largely sedentary (SED). Black arrow indicates the stratum corneum layer, double asterisk indicates the stratum spinosum, and single asterisk indicates the dermis. (C) Stratum spinosum layer thickness in SED and ACT subjects in various age groups. (D) Reticular dermis collagen content in each age group and (E) representative trichrome-stained cross sections of human skin. Data from SED and ACT groups have been combined as there was not a group effect. (F) mtDNA copy number in skin and (G) buccal cell samples from habitually sedentary (SED) and highly active (ACT) individuals. (H) mRNA expression of *Cox7b,* (I) *Nd1*, and (J) *Pgc-1*α in basal buccal cell samples from 20- to 86-year old SED and ACT subjects. Data are mean ± SE. For all analyses, *n *= 11–19 per age and activity group. Cross-sectional analyses and comparisons were made using a two-way anova. *indicates a significant (*P *<* *0.05) difference from the indicated group(s). ^#^indicates a significant (*P *<* *0.05) overall activity group effect. ^†^indicates a difference (*P *<* *0.05) from 20–39 year old individuals.

Exercise robustly alters whole-body metabolism and protects from age-associated physical deterioration and disease, although the most well-established positive adaptations involve metabolic tissues such as liver, skeletal muscle, or the cardiovascular system (Lanza *et al*., 2008; Seals *et al*., 2009). To determine whether parallel adaptations occur in skin, we assessed changes in skin structure and physiology in habitually active subjects (ACT, ≥4 h week^−1^ of high-intensity aerobic exercise) compared to sedentary controls (SED, ≤1 h week^−1^ of exercise) across the human lifespan. Importantly, these subjects were recruited based on their regular duration of physical activity and not their performance to make our conclusions more relevant to the general population. We found ACT subjects had a thinner stratum corneum epidermal layer throughout the lifespan (Fig.1A,B; for subject characteristics see Table S1). Additionally, the oldest (65–86 years) ACT subjects also had attenuated thinning of the stratum spinosum layer of the epidermis compared to the SED subjects (Fig.1C). However, habitual exercise did not appear to influence the age-related loss of reticular dermis collagen (Fig.1D,E). As others have observed similar improvements to the skin of aged mice using interventions that enhance mitochondrial biogenesis (Dillon *et al*., 2012), we assessed mtDNA content and gene expression in ACT and SED subjects in skin as well as commonly available buccal cell (cheek swab) samples. We found that mtDNA copy number in buccal cells and skin was greater in ACT vs. SED subjects (Fig.1F,G). Similarly, basal levels of buccal cell mitochondrial genes were only maintained with age in ACT subjects (Fig. 1H–J). These findings indicate that exercise can attenuate some aspects of skin aging in humans and that these changes are associated with improvements in tissue mitochondria.

**Fig 2:**
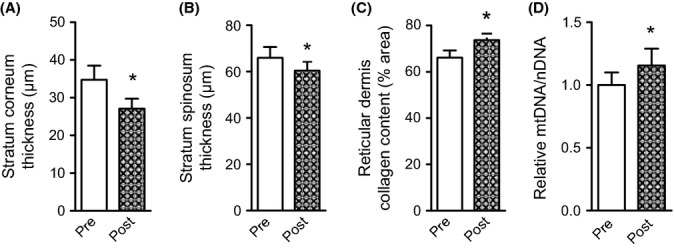
A short-term aerobic exercise intervention improves skin structure and increases tissue mitochondria. (A) Stratum corneum thickness, (B) stratum spinosum thickness (C) reticular dermis collagen content, and (D) mtDNA copy number in skin samples acquired before (Pre) and after 3 months of aerobic exercise training (Post) in previously sedentary elderly (65–86 year) adults. Data are mean ± SE. *n *=* *10. Pre/post-training comparisons were made using a paired *t*-test. *indicates a significant (*P *<* *0.05) difference from the indicated group(s).

As our cross-sectional comparisons could be influenced by unknown confounding variables across the study groups, we chose to enroll a subset of the sedentary elderly adults into a 3-month cycling exercise program to more directly determine the ability of exercise to reverse age-related changes to human skin. We found that 12 weeks of endurance exercise training in sedentary elderly adults reduced stratum corneum thickness (Fig.2A) and, unexpectedly, decreased stratum spinosum thickness (Fig.2B). This contrasting effect of exercise in the stratum spinosum compared to our cross-sectional cohort may be due to our use of a relatively short exercise intervention, whereas a longer intervention may promote more extensive remodeling that could restore stratum spinosum thickness. Additionally, endurance exercise training in elderly SED subjects increased collagen content (Fig.2C) and skin mtDNA copy number (Fig.2D).

**Fig 3:**
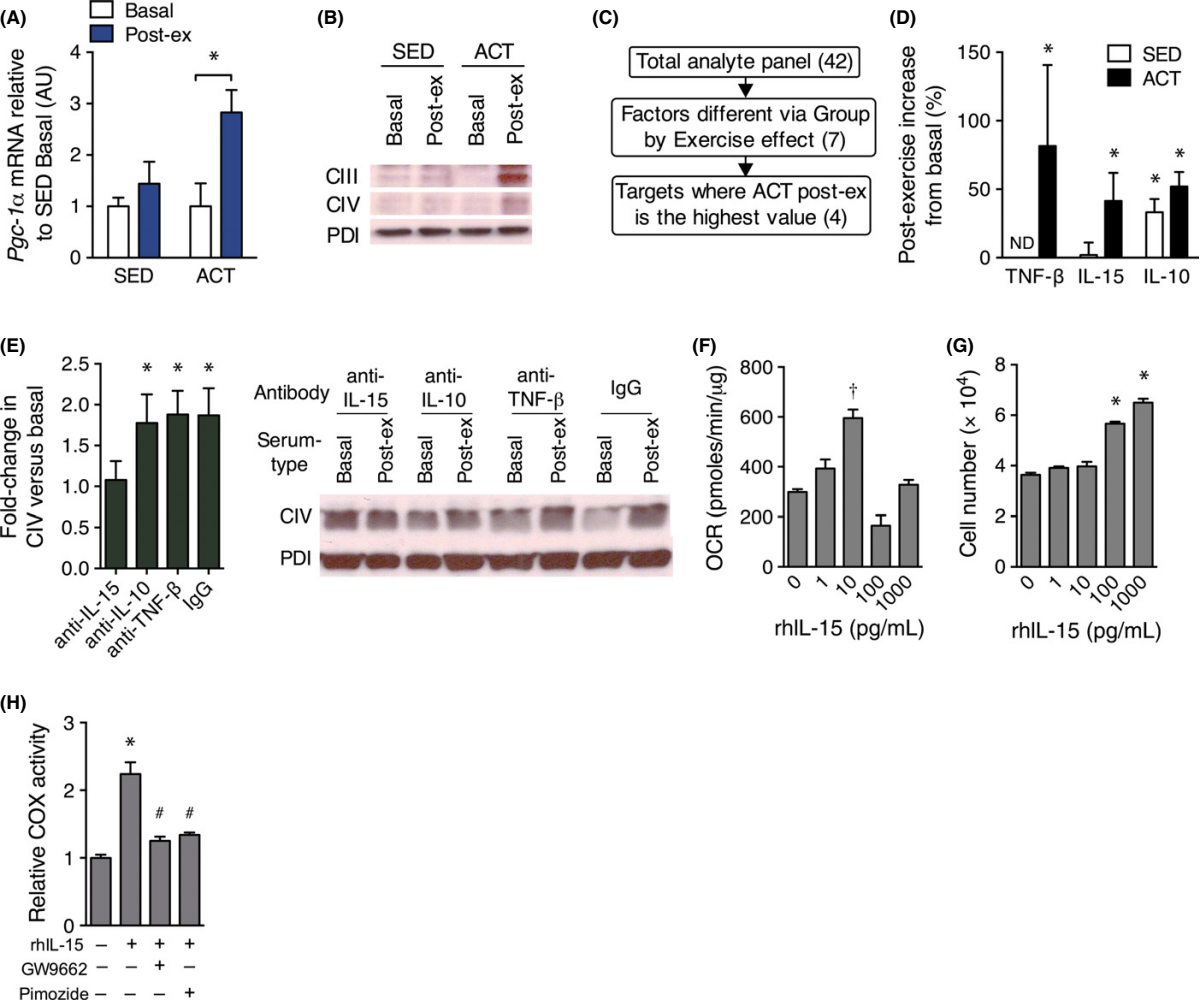
IL-15 is responsible for exercise-stimulated mitochondrial biogenesis in skin *in vitro*. (A) Expression of *Pgc-1*α mRNA in buccal swabs prior to and immediately following a single session of aerobic exercise using *Gapdh* as a stable housekeeping gene. *n *= 8 per group. (B) Representative immunoblots of mitochondrial protein subunits of complex III (core 2, CIII) and complex IV (COX II, CIV) in primary dermal fibroblasts that were incubated for 48 h with media containing 10% human serum from the indicated blood sample. *n *=* *4 replicates per condition. (C) Criteria used to analyze the results of a plasma cytokine panel for determination of factors induced by exercise that are likely to signal peripheral tissue mitochondrial biogenesis. Numbers in parentheses indicate the number of analytes that pertain to the successive criteria. (D) The percent change in plasma IL-15, IL-10, and TNF-β in SED and ACT groups in response to a single bout of exercise. *n *=* *8 per group. (E) Immunoblots of mitochondrial complex IV protein in primary dermal fibroblasts incubated for 48 h in enriched media containing 10% basal or postexercise (Post-ex) serum from ACT subjects that was pretreated with control IgG, anti-TNF-β, anti-IL-15, or IL-10-neutralizing antibodies. *n *=* *4 replicates per condition. (F) Oxygen consumption rate (OCR) and (G) cell counts of human primary dermal fibroblasts treated with the indicated concentrations of rhIL-15 for 48 h. *n *=* *3. (H) COX activity in dermal fibroblasts that were untreated (CON) or incubated with 10 pg mL^−1^ rhIL-15 with and without 1 μm GW9662 or 5 μm pimozide for 48 h. Data are mean ± SE. Data in A and D were compared using a two-way repeated-measures anova. Data in E were compared using a paired *t*-test. Data in F–H were compared using a one-way anova. *P <* *0.05 from basal or control condition. ^†^*P *<* *0.05 from all other conditions. ^#^*P *<* *0.05 from rhIL-15 only condition. ND, not detectable.

To determine whether ACT individuals produced a unique mitochondrial response to exercise, we measured the expression of the master regulator of mitochondrial biogenesis, *Pgc-1*α*,* in buccal cells from each group immediately after exercise and found that only ACT subjects increased *Pgc-1*α expression postexercise (Fig.3A). We hypothesized that a circulating factor was mediating our observed changes in mitochondria; therefore, we incubated human primary dermal fibroblasts in media conditioned with 10% human serum acquired from young SED and ACT individuals prior to and following acute exercise. Similar to our findings regarding buccal swab *Pgc-1*α mRNA, we found that only ACT postexercise serum-conditioned media increased mitochondrial content in fibroblasts (Fig.3B), an effect that was completely ablated when serum proteins were precipitated from solution (Fig. S1A,B). We then evaluated plasma samples from these subjects before and after exercise using a panel of known cytokines and chemokines to screen for proteins that were uniquely altered in the ACT postexercise condition (Fig.3C, complete results in Table S2). This analysis produced four candidate proteins: TNF-β, IL-15, IL-10, and RANTES; however, as RANTES has only been associated with inflammation, we focused on the remaining analytes (Figs3D and S1C). We then tested which of these analytes were essential to the mitochondrial response by pretreating the serum with neutralizing antibodies to TNF-β, IL-10, or IL-15 and found that only anti-IL-15 antibodies mitigated the exercise-stimulated increase in mitochondrial complex IV (Fig.3E) and citrate synthase activity (Fig. S1D) in the fibroblasts. We next determined whether IL-15 stimulates mitochondrial function in a dose-dependent and cell-autonomous manner by measuring mitochondrial respiration in human dermal fibroblasts treated with recombinant human IL-15 (rhIL-15) between 0 and 1000 pg mL^−1^. Mitochondrial respiration increased with rhIL-15 doses up to 10 pg mL^−1^, then declined (Fig.3F), suggesting a narrow window of therapeutic effectiveness that corresponds closely to our observed postexercise levels of IL-15 in human plasma (5.6 ± 1.2 pg mL^−1^). Higher rhIL-15 levels of 100 and 1000 pg mL^−1^ of rhIL-15 caused an increase in dermal fibroblast proliferation vs. control (Fig.3G), similar to previously described growth effects in liver (Suzuki *et al*., 2006). Additionally, when we co-incubated primary human fibroblasts with the rhIL-15 and PPARγ antagonist GW9662 or the STAT5 inhibitor pimozide, the increase in cytochrome *c* oxidase (COX) activity due to rhIL-15 was ablated (Fig.3H). These data are consistent with previous work showing a mitigation of progeroid aging due to mtDNA mutations in skin using the PPARγ agonist bezafibrate (Dillon *et al*., 2012) as well as a requirement for both STAT5 and PPARγ in keratinocyte differentiation (Dai *et al*., 2007).

**Fig 4:**
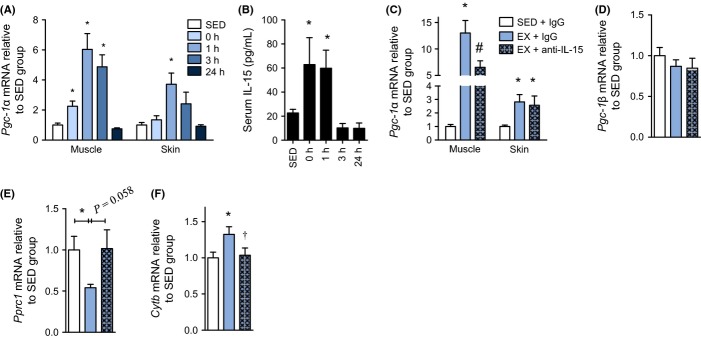
IL-15 partially regulates exercise-mediated mitochondrial signaling in skin and skeletal muscle. (A) qPCR of *Pgc-1*α mRNA in tissues from wild-type mice that were sacrificed at the indicated times after cessation of exercise or that did not exercise (SED). Muscle is *quadriceps* tissue, *n *=* *9 per group. (B) Corresponding serum IL-15 concentration at the indicated times postexercise. *n *=* *9 per group. (C) *Pgc-1*α mRNA expression in *quadriceps* muscle and skin from mice that remained sedentary or exercised in conjunction with injection of IgG control or anti-IL-15-neutralizing antibody. *n *=* *6–10 per group. (D) *Pgc-1*β and (E) *Pprc1* mRNA expression in skin tissue from mice that remained sedentary or exercised in conjunction with injection if IgG control or anti-IL-15-neutralizing antibody. *n *=* *6–10 per group. (F) *Cytb* mRNA in skin acquired from wild-type mice that rested (SED) or were subjected to treadmill exercise in conjunction with tail vein injection of a total of 5 μg of IgG control or anti-IL-15 antibody. *n *=* *6–10 per group. Mice in the neutralization experiments were injected with 2.5 μg antibody immediately before and after the exercise bout and sacrificed at 1 h following cessation of exercise. All acute mouse exercise experiments were performed at a 10-degree uphill grade at a speed of 16 m per minute for 1 h. mRNA expression analyses used β2-microglobulin as a stable housekeeping gene. Data are mean ± SE. Data were compared using a one-way anova. *P <* *0.05 from SED group. ^†^*P *<* *0.05 from EX + IgG. ^#^*P *<* *0.05 from all other conditions.

Exercise improves skeletal muscle mitochondrial capacity via signaling cascades that promote mitochondrial biogenesis (O’Neill *et al*., 2013). We sought to determine whether a regulator of mitochondrial biogenesis, *Pgc-1*α*,* was elevated in skin following exercise. We found that a single session of treadmill running in mice caused a transient elevation in *Pgc-1*α mRNA in both skeletal muscle and skin, peaking at 1 h postexercise (Fig.4A) in parallel to the rise in serum IL-15 (Fig.4B). To determine whether IL-15 was necessary for exercise-stimulated mitochondrial biogenesis *in vivo*, we exercised wild-type mice that were given a venous injection of IL-15-neutralizing antibody or IgG control prior to and following 1 h of treadmill exercise. We found that neutralizing circulating IL-15 partially reduced the exercise-induced elevation in skeletal muscle *Pgc-1*α expression, but not skin *Pgc-1*α expression (Fig.4C). Skin *Pgc-1*β expression was also not affected by the IL-15-neutralizing antibody (Fig.4D). However, IL-15 neutralization prevented an exercise-stimulated reduction in skin PPARγ coactivator-related protein 1 (*Pprc1*) mRNA (Fig. S4E) and importantly abolished an increase in downstream mitochondrial gene expression of cytochrome *b* (Fig.4F). These results suggested that IL-15 partially mediates exercise-stimulated mitochondrial biogenesis in skin and skeletal muscle.

**Fig 5:**
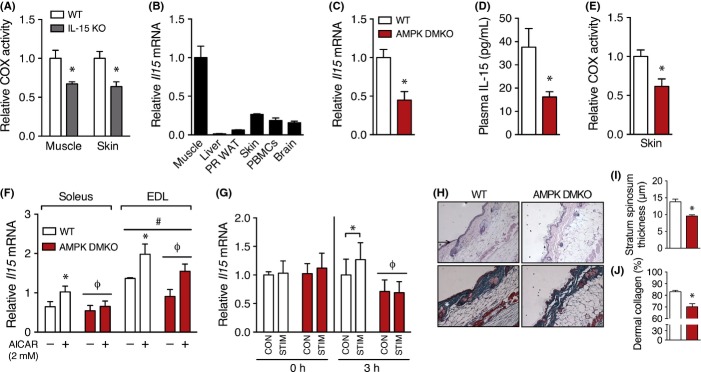
IL-15 expression and skin metabolism are regulated by skeletal muscle AMPK. (A) COX activity relative to WT mice in skeletal muscle (*quadriceps*), and skin tissue from WT and IL-15 KO mice. *n *=* *9. (B) Relative *Il15* mRNA expression in body tissues. *n *=* *8–10. (C) *Il15* mRNA in *gastrocnemius* muscle (*n *=* *4) and (D) plasma IL-15 levels in 3-month-old wild-type littermates (WT) and AMPK β1β2 double-muscle knockout (AMPK DMKO) mice (*n *=* *5). (E) Cytochrome *c* oxidase activity in skin from WT and AMPK DMKO mice. (F) *Il15* mRNA expression in isolated *EDL* and *soleus* muscles from WT and AMPK DMKO mice that were incubated with 2 mm AICAR or vehicle for 2 h. *n *=* *3. (G) *Il15* mRNA expression in *tibialis anterior* muscle from WT and AMPK DMKO mice that were subjected to a 30-min *in situ* electrical stimulation protocol (STIM) or served as the contralateral control (CON). *n *=* *4–5. (H) Histological images of skin from 18-month-old wild-type and AMPK DMKO littermates stained with H&E (top) or trichrome (bottom). Black arrowhead is the stratum spinosum and the white asterisk indicates the dermis. (I) Quantification of skin dermal collagen and (J) stratum spinosum thickness in aged WT and AMPK DMKO mice. *n *=* *5. mRNA expression was normalized to *Gapdh* or β*-actin* as a stable housekeeping gene. Results in A, C–E, and I–J were compared using an unpaired *t*-test. Data in F were analyzed using a three-way anova. G was compared using two-way repeated-measures anova. *Significantly different (*P *<* *0.05) from the indicated group or from the control condition. ^ϕ^Main effect of genotype. ^#^Main effect of muscle type. Data are mean ± SE.

Next, we sought to determine whether IL-15 was required for basal mitochondrial function, so we evaluated tissue mitochondria in mice lacking whole-body expression of IL-15 (IL-15 KO) and found lower COX activity in skin and skeletal muscle tissue from IL-15 KO mice (Fig.5A). IL-15 is known to be highly expressed in skeletal muscle (Pedersen & Febbraio, 2012) and also secondarily in skin when compared across metabolic tissues (Fig.5B). As acute exercise capacity was predictive of the positive effects on skin tissue in our human subjects (20–39 year VO_2_ peak, SED: 34 ± 2 mL kg^−1^ min^−1^, ACT: 57 ± 2 mL kg^−1^ min^−1^), this implicated the involvement of muscle AMP-activated protein kinase (AMPK), a well-described energy-sensing molecule that regulates exercise capacity (O’Neill *et al*., 2011). We tested whether AMPK was involved in the expression and/or induction of IL-15 via exercise using mice that lacked both the β1- and β2-subunits of AMPK in skeletal muscle (AMPK DMKO). These mice have exercise intolerance due to reduced muscle mitochondria and impaired contraction-stimulated glucose uptake but appear phenotypically normal at rest compared to wild-type littermates (O’Neill *et al*., 2011). We found that muscle *Il15* mRNA expression and plasma IL-15 were reduced in AMPK DMKO mice (Fig.5C,D) and, similar to the IL-15 KO mice, AMPK DMKO mice had reduced COX activity in skin tissue (Fig.5E). To further explore whether muscle AMPK was necessary for the stimulation of *Il15* expression, we incubated skeletal muscle from WT and AMPK DMKO mice *ex vivo* in the presence or absence of the AMPK activator AICAR. AICAR produced an increase in *Il15* in both soleus and EDL muscles in wild-type, but not in AMPK DMKO mice (Fig.5F). To mimic exercise, we examined *Il15* expression in *tibialis anterior* (TA) muscles following a 30-min contraction protocol, matching the work performed by each group (average force of contraction – WT: 236 ± 11 mN, AMPK DMKO: 232 ± 10 mN). We found no changes in *Il15* mRNA were present at the 0 h time point, but that *Il15* expression was specifically increased in wild-type but not in AMPK DMKO mice 3 h after the muscle contractions compared to the resting limb (Fig.5G), indicating that contraction-stimulated *Il15* expression is dependent on AMPK activity. Furthermore, we analyzed skin morphology in older (18 month old) WT and AMPK DMKO mice and found reduced collagen content (Fig.5H,I) and reduced dermal thickness in AMPK DMKO mice (Fig.5H,J), consistent with their skin mitochondrial defects. Overall, these results suggest that IL-15 is regulated by muscle AMPK and that skeletal muscle metabolism can regulate skin morphology.

**Fig 6:**
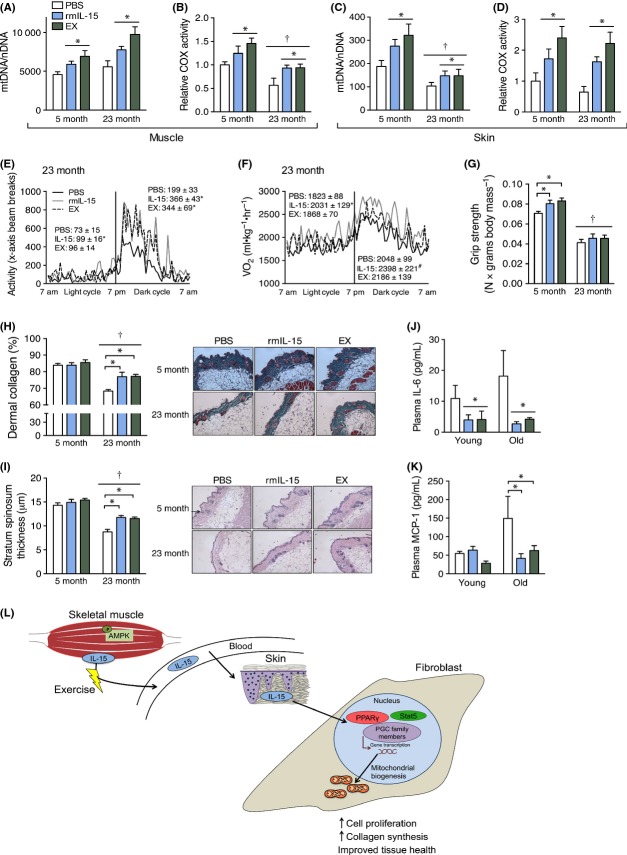
Daily IL-15 therapy in mice mimics the anti-aging effects of exercise on skin structure and mitochondria. Chow-fed mice were injected once daily via tail vein with PBS, recombinant mouse IL-15 (rmIL-15), or underwent forced exercise training (EX) for 33 consecutive days and were sacrificed at the age indicated. (A) *Quadriceps* muscle mtDNA copy number and (B) cytochrome *c* oxidase (COX) activity. *n *=* *6–8 per group. (C) Skin mtDNA copies and (D) COX activity. *n *=* *6–8 per group. (E) Cage activity and (F) oxygen uptake (VO_2_) over a 24-h period in 23-month-old mice treated with PBS, rmIL-15, or EX. (G) Peak grip strength in each treatment group normalized to body weight. (H) Quantification of dermal collagen content (left) and representative images of trichrome-stained skin cross sections from each treatment group (right). *n *=* *4–8 per group. White asterisk indicates the dermis. (I) Quantification of stratum spinosum thickness and representative images of H&E-stained cross sections of skin from each group. *n *=* *4–8 per group. Arrow indicates the stratum spinosum layer. Scale bar is 100 μm. (J) Plasma IL-6 and (K) MCP-1 drawn at sacrifice from mice in each treatment group. (L) Graphical illustration of the effects of exercise-induced muscle IL-15 signaling to skin tissue. Five-month-old and 23-month-old mice were injected with 500 and 1000 pg of rmIL-15, respectively. All data were compared using a two-way anova. *indicates a significant difference (*P *<* *0.05) from PBS or the indicated group. ^†^indicates an overall effect of age. Data are mean ± SE.

As IL-15 appears to regulate exercise-stimulated mitochondrial signaling in skin and skeletal muscle, we sought to clarify its therapeutic potential in young (5 month) and old (23 month) mice with daily intravenous injections of recombinant mouse IL-15 (rmIL-15) that mimicked the physiologic elevation of endogenous IL-15 observed following acute exercise (Fig. S2A). Both young and old mice that received rmIL-15 injections or exercise had significantly higher skin and muscle COX activity, and mtDNA copy number (Fig.6A–D). However, in contrast to previous findings from muscle IL-15-overexpressing mice (Quinn *et al*., 2009), there was no change in body or tissue mass in any of the groups (Fig. S2B–F) and only the exercise group had an increase in treadmill running capacity (Fig. S2G). Moreover, oxygen consumption, cage movement, and food intake were similar among 5-month-old mice (Fig. S3A–C), indicating that behavioral changes did not account for the changes in mitochondria. However, both rmIL-15 and exercise treatment resulted in higher cage activity and oxygen use in 23-month-old mice (Fig.6E,F), indicating a partial restoration of physical function specifically in the aged mice, although there was no difference in food consumption (Fig. S3C). Despite no change in muscle mass, rmIL-15 and exercise treatment also improved muscle grip strength in 5-month-old mice (Fig.6G). Finally, rmIL-15 and exercise treatment resulted in higher stratum spinosum thickness and dermal collagen content in 23-month-old mice compared with PBS-treated mice, which partially reversed the effects of aging (Fig.6H,I). These findings are in agreement with reports showing stimulation of collagen production *in vitro* by rhIL-15 (Kim *et al*., 2014) and PPAR agonists (Ham *et al*., 2010) and demonstrate that physiologic elevations of circulating IL-15 can mimic some of beneficial effects of exercise training on aging skin and skeletal muscle metabolism. Furthermore, as inflammation is a hallmark of aging and metabolic dysfunction (Green *et al*., 2011), we measured plasma cytokines from each treatment group and found that rmIL-15 and exercise treatment reduced circulating levels of the inflammatory cytokines IL-6 and MCP-1 (Fig6J,K).

## Discussion

The broad, systemic benefits of exercise on the aging process (Chakravarty *et al*., 2008) are likely complexly regulated among many organs. We identify circulating IL-15 as an exercise-stimulated hormone that mediates the health of skin tissue. While IL-15 is one of many factors secreted from skeletal muscle that is elevated following exercise in mice and humans (Pedersen & Febbraio, 2012), the regulation of muscle-derived IL-15 and its physiological consequences has remained unclear. IL-15 has been shown to be elevated in humans after exercise in some studies (Tamura *et al*., 2011), but not all (Nielsen *et al*., 2007). The relatively transient increase in IL-15 in circulation immediately following exercise seen in the current work as well as the work of Tamura *et al*. (2011), suggests that the timing of blood sampling is important in detecting the exercise response. Moreover, an immediate change in circulating levels of IL-15 suggests a pool of readily secretable IL-15 that would not necessarily be reliant on transcriptional changes in IL-15 mRNA, as noted by others (Bamford *et al*., 1998).

Muscle IL-15 has been previously associated with whole-body metabolic changes. Work using muscle IL-15-overexpressing mice has suggested that IL-15 negatively regulates adiposity (Quinn *et al*., 2009), enhances muscle mass, and improves exercise capacity; however, these mice are hyperactive at a young age (Quinn *et al*., 2009), which may account for much of their improved energy metabolism. Additionally, as circulating IL-15 is ∼5000-fold higher in these mice compared to our basal levels, their effects ascribed to IL-15 may not be physiologically relevant. In particular, we see an increase in muscle strength in young mice, but no changes in muscle mass with rmIL-15 treatment, possibly due to our use of a much lower dose than the mouse muscle overexpression model. However, it is possible that a more prolonged treatment period with low-dose rmIL-15 would have produced more robust changes in muscle size in light of the fact that a splice variant of *Pgc-1*α has been shown to enhance muscle mass (Ruas *et al*., 2012).

IL-15 is necessary for natural killer immune cell activation and expansion in response to viral pathogens, primarily through the IL-15 receptor βγ complex (Waldmann, 2006). IL-15 binds to its β- and γ-receptors (Kd: 10^–9^ m) (Giri *et al*., 1994) but has higher affinity for its α-receptor (Kd: 10^–11^ m) (Anderson *et al*., 1995), indicating that expression well below 10^–9^ m could avoid overt pathogen-like immune system activation. Exercise and our injected dose of IL-15 in wild-type mice were suitable to transiently reach a peak of ∼100 pg mL^−1^ (∼0.76 × 10^−11^ m) in circulation, possibly low enough to avoid the negative consequences of chronically elevated IL-15 that may trigger leukemia (Fehniger *et al*., 2001) or pancreatic β-cell death (Chen *et al*., 2013) in mice. IL-15 has been administered to mice (1200–7200 μg kg^−1^) (Munger *et al*., 1995) and rhesus macaques (10–50 μg kg^−1^) (Waldmann *et al*., 2011) at far higher doses than our injected dose (∼17 ng kg^−1^). However, the ability of doses of IL-15 higher than those experienced during exercise to mediate changes in skin mitochondrial function remains questionable, as levels higher than 10 pg mL^−1^ did not stimulate mitochondrial respiration in dermal fibroblasts.

We hypothesize that fewer ‘pulses’ of IL-15 from skeletal muscle and other tissues during sedentary living are in part responsible for the accelerated degeneration of dermal fibroblasts that drive the clinical symptoms of skin aging. These findings are bolstered by prior reports that mitochondrial energetics play a central role in aging (Trifunovic *et al*., 2004) and that mitochondrial biogenesis can retard cellular aging (Sahin & Depinho, 2010; Safdar *et al*., 2011). Moreover, the finding that muscle AMPK deletion causes skin deterioration is in line with previous work, showing that the circulating environment can strongly affect brain and muscle aging (Villeda *et al*., 2011; Katsimpardi *et al*., 2014; Sinha *et al*., 2014). As low muscle AMPK activity is commonly observed in a wide range of health disorders (Steinberg & Jørgensen, 2007), reduced basal or exercise-stimulated IL-15 secretion and other AMPK-dependent factors may exacerbate skin or muscle tissue disease progression.

As mitochondrial degeneration is widely implicated in disease, there are many potential conditions where the exercise mimicking effects of IL-15 therapy might be useful, such as in frail patients or those with metabolic disease that have a low exercise capacity. Additionally, IL-15 may prove beneficial for cutaneous wound healing as stimulating mitochondrial metabolism improves skin responses to injury (Ham *et al*., 2010). Given the prior findings that IL-15 may stimulate browning of white adipose tissue (Boström *et al*., 2012) and promote liver repair (Suzuki *et al*., 2006), exercise-induced IL-15 may also be responsible for improvements in other organs and prove a multifaceted strategy to treat health disorders.

## Experimental procedures

### Human subject recruitment and testing

A subset of the human subjects in this study have been previously described (Crane *et al*., 2013b), and all experimental procedures were approved by the institutional research ethics board. Tissue samples from human subjects were collected following an overnight fast. Skin samples were acquired from the upper portion of the non-sun-exposed buttocks below the waistline using a 4-mm punch biopsy under local anesthetic without norepinephrine. Subcutaneous fat was separated from the bottom portion of the dermis following tissue collection. Buccal swabs and plasma and serum samples were collected at rest and immediately following acute exercise. All samples were flash-frozen in liquid nitrogen. After baseline tissue collections, subjects underwent a bout of exercise testing to confirm aerobic fitness, as described (Crane *et al*., 2013b). Within 5 min of finishing, each subject then underwent 30 min of exercise at 50% of their cycling power maximum. In total, the subjects cycled for approximately 45 min each.

In addition to the acute exercise trial, a portion of the 65-86 year old sedentary subjects underwent 12 weeks of twice-weekly aerobic exercise training on a Monark Cardio Care 827E cycle ergometer. The training program started with participants maintaining 65% of HRmax for 30 minutes and progressed by 5% every other week until intensity reached 75% of HR max, at which point the duration of exercise increased by 5 minutes every other week reaching 75% of HR max for 45 minutes at the end of the training intervention.

### Sample analysis

Plasma (EDTA) cytokines from human subjects were initially analyzed using a 42-analyte multiplex ELISA assay (Millipore, Millipore, Billerica, MA, USA), and subsequent mouse IL-15 analyses were performed as a single-plex ELISA. GM-CSF, IFN-γ, IL-10, IL-12 (p70), IL-13, IL-1β, IL-2, IL-4, IL-5, IL-6, IL-7, IL-8, and TNF-α were assessed using high-sensitivity ELISAs (0.13–2000 pg mL^−1^), and all other analytes were analyzed using standard detection limits (3.2–10 000 pg mL^−1^). Proteins in human serum were precipitated using ethanol, followed by centrifugal pelleting of the precipitant and removal of the supernatant that contained soluble metabolites. The supernatant fraction was then dried using a vacuum centrifuge, and the metabolites were resuspended in sterile water to the original serum volume. The removal of all proteins was confirmed using a protein assay, and this fraction was added to intact serum for cell culture experiments as indicated. Primary human dermal fibroblasts were cultured according to standard explant methods (Villegas & McPhaul, 2005) and all cell treatments were for 48 h. The recombinant human IL-15 was purchased from R&D Systems, and the GW9662 compound and pimozide were acquired from Sigma-Aldrich Sigma-Aldrich, St. Louis, MO, USA Abcam, Cambridge, UK R&D Systems, Minneapolis, MN, USA.

Cell and tissue lysates were prepared using 0.05 m potassium phosphate buffer, and mitochondrial protein immunoblotting was performed as described using human or rodent antibodies from Abcam (#ab110411, #ab110413). Cytochrome *c* oxidase activity was performed by incubating sample lysates with reduced cytochrome *c* and measuring the change in absorbance at 550 nm over 90 s in a 96-well plate. DNA and RNA isolation and qPCR of tissues were performed as described (Crane *et al*., 2013a).

All mouse experiments were performed using female mice on a chow diet. IL-15 knockout mice (IL-15 KO) and C57/BL6 control mice were obtained from Taconic and sacrificed at 12 weeks of age. Wild-type mice for acute exercise experiments were obtained from Jackson laboratories and sacrificed at 16 weeks of age. AMPK DMKO mice, and wild-type littermates were bred and housed in the McMaster University Animal Facility under standard housing conditions with a 12-h light/dark cycle. Acute exercise experiments were performed for 1 h at a speed of 16 m per minute at an uphill grade of 10 degrees. All neutralizing antibodies were purchased from R&D Systems.

### *Ex vivo* and *in situ* muscle experiments

Soleus and EDL muscles were excised under anesthesia from AMPK DMKO mice and wild-type littermates, incubated in 1 mL of media at 30 °C for 2 h with and without AICAR before being removed, and snap-frozen. *In situ* contraction experiments were performed by first isolating the tibialis anterior (TA) muscle under anesthesia (ketamine/xylazine) from both legs and connecting the distal tendon of one leg to a force transducer with string. The sciatic nerve of that leg was then exposed and enervated using pulsed electrical stimulation 400 time at 1.5–4 milliamps at a frequency of 10 Hz for 0.5 s over the course of 30 min and the contralateral leg served as a nonstimulated control. Mice were maintained on a 33 °C peltier warmed block under isofluroane gas anesthetic during the contraction and recovery periods using a carrier gas of 95% oxygen. TA muscles were harvested immediately following (0 h) the contraction protocol or after 3 h.

### Animal testing

Grip strength was tested in mice as described (Ogborn *et al*., 2012). Mouse behavior (cage activity, food intake) and metabolic parameters (VO_2_, VCO_2_, RER) were analyzed using Columbus Lab Animal Monitoring System (CLAMS; Columbus instruments, Columbus, OH, USA) over a 24-h period with lights on at 07:00 and off at 19:00 hours.

C57/BL6 mice that were 4 and 22 months old were randomly allocated to vehicle (PBS), rmIL-15, or exercise daily treatment for 33 consecutive days. rmIL-15 (5-month-old group: 500 pg; 23-month-old group: 1000 pg) or vehicle control (PBS) was injected via the tail vein using a tuberculin syringe. This dosing was used based on pilot work in 3- to 4-month-old mice (Fig. S3A) and because the 23-month-old mice weighed approximately twice as much as the 5-month-old mice at the start of the experiment.

Exercise-trained mice underwent 33 consecutive days of progressive forced treadmill running. The first week of exercise training occurred at a treadmill speed of 16 m per minute for 5-month-old mice and 10 m per minute for 23-month-old mice at a 10-degree uphill grade for 1 h. After one week, the 23-month-old group was increased to 12 m per minute and after 2 weeks increased to 14 m per minute. After the first week, 5-month-old mice were increased to 18 m per minute for the remainder of the training period. Grade and duration remained unchanged for the training period.

## Supporting Information

**Fig. S1.**Exercise serum deproteinization and plasma analyte screen results. **Fig. S2.** Recombinant mouse IL-15 dosing and body and tissue weights from daily PBS, rmIL-15 and EX treatments. **Fig. S3.** Metabolic and behavioral characteristics of 5 month old treated mice. **Table S1.** Subject physical characteristics. **Table S2**. Results of the exercise-stimulated plasma cytokine and chemokine screen in young individuals.

## References

[b1] Anderson DM, Kumaki S, Ahdieh M, Bertles J, Tometsko M, Loomis A, Giri J, Copeland NG, Gilbert DJ, Jenkins NA (1995). Functional characterization of the human interleukin-15 receptor alpha chain and close linkage of IL15RA and IL2RA genes. J. Biol. Chem.

[b2] Bamford RN, DeFilippis AP, Azimi N, Kurys G, Waldmann TA (1998). The 5’ untranslated region, signal peptide, and the coding sequence of the carboxyl terminus of IL-15 participate in its multifaceted translational control. J. Immunol.

[b3] Boström P, Wu J, Jedrychowski MP, Korde A, Ye L, Lo JC, Rasbach KA, Bostrom EA, Choi JH, Long JZ, Kajimura S, Zingaretti MC, Vind BF, Tu H, Cinti S, Hojlund K, Gygi SP, Spiegelman BM (2012). A PGC1-α- dependent myokine that drives brown-fat-like development of white fat and thermogenesis. Nature.

[b4] Chakravarty EF, Hubert HB, Lingala VB, Fries JF (2008). Reduced disability and mortality among aging runners: a 21-year longitudinal study. Arch. Intern. Med.

[b5] Chen J, Feigenbaum L, Awasthi P, Butcher DO, Anver MR, Golubeva YG, Bamford R, Zhang X, St Claire MB, Thomas CJ, Discepolo V, Jabri B, Waldmann TA (2013). Insulin-dependent diabetes induced by pancreatic beta cell expression of IL-15 and IL-15Rα. Proc. Natl Acad. Sci. USA.

[b6] Coppé J-P, Patil CK, Rodier F, Sun Y, Muñoz DP, Goldstein J, Nelson PS, Desprez P, Campisi J (2008). Senescence- associated secretory phenotypes reveal cell-nonautonomous functions of oncogenic RAS and the p53 tumor suppressor. PLoS Biol.

[b7] Crane JD, Abadi A, Hettinga BP, Ogborn DI, Macneil LG, Steinberg GR, Tarnopolsky MA (2013a). Elevated mitochondrial oxidative stress impairs metabolic adaptations to exercise in skeletal muscle. PLoS One.

[b8] Crane JD, Macneil LG, Tarnopolsky MA (2013b). Long-term aerobic exercise is associated with greater muscle strength throughout the life span. J. Gerontol. A Biol. Sci. Med. Sci.

[b9] Dai X, Sayama K, Shirakata Y, Hanakawa Y, Yamasaki K, Tokumaru S, Yang L, Wang X, Hirakawa S, Tohyama M, Yamauchi T, Takashi K, Kagechika H, Hashimoto K (2007). STAT5a/PPARgamma pathway regulates involucrin expression in keratinocyte differentiation. J. Invest. Dermatol.

[b10] Dillon LM, Hida A, Garcia S, Prolla TA, Moraes CT (2012). Long-term bezafibrate treatment improves skin and spleen phenotypes of the mtDNA mutator mouse. PLoS One.

[b11] Fehniger TA, Suzuki K, Ponnappan A, VanDeusen JB, Cooper MA, Florea SM, Freud AG, Robinson ML, Durbin J, Caligiuri MA (2001). Fatal leukemia in interleukin 15 transgenic mice follows early expansions in natural killer and memory phenotype CD8 +  T cells. J. Exp. Med.

[b12] Fisher GJ, Kang S, Varani J, Bata-Csorgo Z, Wan Y, Datta S, Voorhees JJ (2002). Mechanisms of photoaging and chronological skin aging. Arch. Dermatol.

[b13] Giri JG, Ahdieh M, Eisenman J, Shanebeck K, Grabstein K, Kumaki S, Namen A, Park LS, Cosman D, Anderson D (1994). Utilization of the beta and gamma chains of the IL-2 receptor by the novel cytokine IL-15. EMBO J.

[b14] Green DR, Galluzzi L, Kroemer G (2011). Mitochondria and the autophagy- inflammation-cell death axis in organismal aging. Science.

[b15] Ham SA, Kim HJ, Kim HJ, Kang ES, Eun SY, Kim GH, Park MH, Woo IS, Kim HJ, Chang KC, Lee JH, Seo HG (2010). PPARdelta promotes wound healing by up-regulating TGF-beta1-dependent or -independent expression of extracellular matrix proteins. J. Cell Mol. Med.

[b16] Harman D (1972). The biologic clock: the mitochondria?. J. Am. Geriatr. Soc.

[b17] Isobe K, Ito S, Hosaka H, Iwamura Y, Kondo H, Kagawa Y, Hayashi JI (1998). Nuclear- recessive mutations of factors involved in mitochondrial translation are responsible for age-related respiration deficiency of human skin fibroblasts. J. Biol. Chem.

[b18] Katsimpardi L, Litterman NK, Schein PA, Miller CM, Loffredo FS, Wojtkiewicz GR, Chen JW, Lee RT, Wagers AJ, Rubin LL (2014). Vascular and neurogenic rejuvenation of the aging mouse brain by young systemic factors. Science.

[b19] Kim M-S, Song HJ, Lee SH, Lee CK (2014). Comparative study of various growth factors and cytokines on type I collagen and hyaluronan production in human dermal fibroblasts. J. Cosmet. Dermatol.

[b20] Lanza IR, Short DK, Short KR, Raghavakaimal S, Basu R, Joyner MJ, McConnell JP, Nair KS (2008). Endurance exercise as a countermeasure for aging. Diabetes.

[b21] Lu CY, Lee HC, Fahn HJ, Wei YH (1999). Oxidative damage elicited by imbalance of free radical scavenging enzymes is associated with large-scale mtDNA deletions in aging human skin. Mutat. Res.

[b22] Munger W, DeJoy SQ, Jeyaseelan R, Torley LW, Grabstein KH, Eisenmann J, Paxton R, Cox T, Wick MM, Kerwar SS (1995). Studies evaluating the antitumor activity and toxicity of interleukin-15, a new T cell growth factor: comparison with interleukin-2. Cell. Immunol.

[b23] Nielsen AR, Mounier R, Plomgaard P, Mortensen OH, Penkowa M, Speerschneider T, Pilegaard H, Pedersen BK (2007). Expression of interleukin-15 in human skeletal muscle effect of exercise and muscle fibre type composition. J. Physiol.

[b24] Ogborn DI, Smith KJ, Crane JD, Safdar A, Hettinga BP, Tupler R, Tarnopolsky MA (2012). Effects of creatine and exercise on skeletal muscle of FRG1-transgenic mice. Can. J. Neurol. Sci.

[b25] O’Neill HM, Maarbjerg SJ, Crane JD, Jeppesen J, Jørgensen SB, Schertzer JD, Shyroka O, Kiens B, van Denderen BJ, Tarnopolsky MA, Kemp BE, Richter EA, Steinberg GR (2011). AMP-activated protein kinase (AMPK) {beta}1{beta}2 muscle null mice reveal an essential role for AMPK in maintaining mitochondrial content and glucose uptake during exercise. Proc. Natl Acad. Sci. USA.

[b26] O’Neill HM, Holloway GP, Steinberg GR (2013). AMPK regulation of fatty acid metabolism and mitochondrial biogenesis: implications for obesity. Mol. Cell. Endocrinol.

[b27] Pedersen BK, Febbraio MA (2012). Muscles, exercise and obesity: skeletal muscle as a secretory organ. Nat. Rev. Endocrinol.

[b28] Quinn LS, Anderson BG, Strait-Bodey L, Stroud AM, Argilés JM (2009). Oversecretion of interleukin-15 from skeletal muscle reduces adiposity. Am. J. Physiol. Endocrinol. Metab.

[b29] Ruas JL, White JP, Rao RR, Kleiner S, Brannan KT, Harrison BC, Greene NP, Wu J, Estall JL, Irving BA, Lanza IR, Rasbach KA, Okutsu M, Nair KS, Yan Z, Leinwand LA, Spiegelman BM (2012). A PGC-1α isoform induced by resistance training regulates skeletal muscle hypertrophy. Cell.

[b30] Safdar A, Bourgeois JM, Ogborn DI, Little JP, Hettinga BP, Akhtar M, Thompson JE, Melov S, Mocellin NJ, Kujoth GC, Prolla TA, Tarnopolsky MA (2011). Endurance exercise rescues progeroid aging and induces systemic mitochondrial rejuvenation in mtDNA mutator mice. Proc. Natl Acad. Sci. USA.

[b31] Sahin E, Depinho RA (2010). Linking functional decline of telomeres, mitochondria and stem cells during ageing. Nature.

[b32] Schroeder P, Gremmel T, Berneburg M, Krutmann J (2008). Partial depletion of mitochondrial DNA from human skin fibroblasts induces a gene expression profile reminiscent of photoaged skin. J. Invest. Dermatol.

[b33] Seals DR, Walker AE, Pierce GL, Lesniewski LA (2009). Habitual exercise and vascular ageing. J. Physiol.

[b34] Sinha M, Jang YC, Oh J, Khong D, Wu EY, Manohar R, Miller C, Regalado SG, Loffredo FS, Pancoast JR, Hirshman MF, Lebowitz J, Shadrach JL, Cerletti M, Kim M, Serwold T, Goodyear LJ, Rosner B, Lee RT, Wagers AJ (2014). Restoring systemic GDF11 levels reverses age-related dysfunction in mouse skeletal muscle. Science.

[b35] Steinberg GR, Jørgensen SB (2007). The AMP-activated protein kinase: role in regulation of skeletal muscle metabolism and insulin sensitivity. Mini Rev. Med. Chem.

[b36] Suzuki A, McCall S, Choi SS, Sicklick JK, Huang J, Qi Y, Zdanowicz M, Camp T, Li Y, Diehl AM (2006). Interleukin-15 increases hepatic regenerative activity. J. Hepatol.

[b37] Tamura Y, Watanabe K, Kantani T, Hayashi J, Ishida N, Kaneki M (2011). Upregulation of circulating IL-15 by treadmill running in healthy individuals: is IL-15 an endocrine mediator of the beneficial effects of endurance exercise?. Endocr. J.

[b38] Treiber N, Maity P, Singh K, Kohn M, Keist AF, Ferchiu F, Sante L, Frese S, Bloch W, Kreppel F, Kochanek S, Sindrilaru A, Iben S, Hogel J, Ohnmacht M, Claes LE, Ignatius A, Chung JH, Lee MJ, Kamenisch Y, Berneburg M, Nikolaus T, Braunstein K, Sperfeld A, Ludolph AC, Briviba K, Wlaschek M, Florin L, Angel P, Scharffetter-Kochanek K (2011). Accelerated aging phenotype in mice with conditional deficiency for mitochondrial superoxide dismutase in the connective tissue. Aging Cell.

[b39] Trifunovic A, Wredenberg A, Falkenberg M, Spelbrink JN, Rovio AT, Bruder CE, Bohlooly-Y M, Gidlof S, Oldfors A, Wibom R, Tornell J, Jacobs HT, Larsson N (2004). Premature ageing in mice expressing defective mitochondrial DNA polymerase. Nature.

[b40] Velarde MC, Flynn JM, Day NU, Melov S, Campisi J (2012). Mitochondrial oxidative stress caused by Sod2 deficiency promotes cellular senescence and aging phenotypes in the skin. Aging (Albany NY).

[b41] Villeda SA, Luo J, Mosher KI, Zou B, Britschgi M, Bieri G, Stan TM, Fainberg N, Ding Z, Eggel A, Lucin KM, Czirr E, Park J, Couillard-Despres S, Aigner L, Li G, Peskind ER, Kaye JA, Quinn JF, Galasko DR, Xie XS, Rando TA, Wyss-Coray T (2011). The ageing systemic milieu negatively regulates neurogenesis and cognitive function. Nature.

[b42] Villegas J, McPhaul M, Ausubel FrederickM (2005). Establishment and culture of human skin fibroblasts. Current Protocols in Molecular Biology.

[b43] Waldmann TA (2006). The biology of interleukin-2 and interleukin-15: implications for cancer therapy and vaccine design. Nat. Rev. Immunol.

[b44] Waldmann TA, Lugli E, Roederer M, Perera LP, Smedley JV, Macallister RP, Godlman CK, Bryant BR, Decker JM, Fleisher TA, Lane HC, Sneller MC, Kurlander RJ, Kleiner DE, Pletcher JM, Figg WD, Yovandich JL, Creekmore SP (2011). Safety (toxicity), pharmacokinetics, immunogenicity, and impact on elements of the normal immune system of recombinant human IL-15 in rhesus macaques. Blood.

[b45] Zouboulis CC, Makrantonaki E (2012). Hormonal therapy of intrinsic aging. Rejuvenation Res.

