# Supplementary material for: Exercise-stimulated interleukin-15 is controlled by AMPK and regulates skin metabolism and aging
Source: Aging Cell. 2015 Apr 22;14(4):625–34. doi: 10.1111/acel.12341 (PMC4531076; doi:10.1111/acel.12341)
Supplement: Supplementary file 1 [file acel0014-0625-sd1.docx]

Supplemental Figure 1


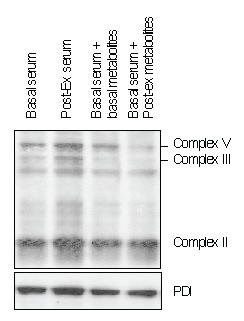
**A** Basal serum **B**

Post-Ex serum

Basal serum + basal metabolites


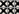


Basal serum + Post-Ex metabolites


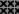


3 # #


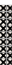

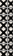

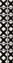

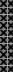

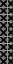

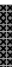


Fold-change of Basal

serum incubation

#

2

1

0

CII CIII CV

**C** Factors different via Group by Exercise interaction (7)

IL-12p40

MDC IL-10

IL-15

TNF-β MIP-1α RANTES

ACT Post-ex is greater than all other samples (4)

IL-10

IL-15

TNF-β

RANTES

**D**

2.0

Fold-change in

CS activity versus basal

1.5

1.0

0.5

0.0

* * *

Supplemental Figure 2

**A B**

5 month

**C**

23 month

100

Serum IL-15 (pg/ml)

40 pg

400 pg

24 PBS 70 rmIL-15

80 23

Body Weight (g)

22

60

21

40 20

20 19

0 18

EX

1 5 9 13 17 21 25 29 33

Day of treatment

60

50

Body Weight (g)

40

30

20

1 5 9 13 17 21 25 29 33

Days of treatment

**D**

5 month

Tissue weight (% body mass)

PBS

4 rmIL-15

**E**

23 month

Tissue weight (% body mass)

8

2.5 EX

2.0

1.5

1.0

0.5

0.0

4

2.0

1.5

1.0

0.5

0.0

**F**

Heart

Tissue weight (% body mass)

0.6

**G**

†

600 #

Total distance run (m)

#

0.4

400

0.2

200

0.0

5 mo. 23 mo.

0

5 mo. 23 mo.

Supplementary Figure 3

**A** 5 month

PBS

Activity (x-axis beam breaks)

**B** 5 month **C**

1500

VO2 (ml•kg-1•hr-1)

Food intake (g•day-1)

rmIL-15

EX

PBS: 644 ± 68 rmIL-15: 685 ± 65

4000 4

1000

500

0

PBS: 110 ± 13 rmIL-15: 108 ± 15

EX: 128 ± 11

EX: 575 ± 41

3000

2000

1000

0

PBS: 2368 ± 43 rmIL-15: 2403 ± 95

EX: 2425 ± 56

3

2

PBS: 3059 ± 34

rmIL-15: 3155 ± 102 1

EX: 3078 ± 70

0

7am

Light cycle

7pm

Dark cycle

7am

7am

Light cycle

7pm

Dark cycle

7am

**Supplementary Table 1.** Subject Physical Characteristics

**20-39 y 40-64 y 65-86 y**

|  | **SED** | **ACT** | **SED** | **ACT** | **SED** | **ACT** | **Age Effect** | **Group Effect** | **Interaction** |
| --- | --- | --- | --- | --- | --- | --- | --- | --- | --- |
| *N* | 15 (6M/9F) | 15 (8M/7F) | 16 (6M/10F) | 22 (15M/7F) | 25 (14M/11F) | 14 (10M/4F) |  |  |  |
| Age (y) | 29±2 | 30±2 | 55±2 | 54±1 | 73±1 | 72±2 | *p* < 0.05 | NS | NS |
| Height (cm) | 169±3 | 176±2 | 167±2 | 175±2 | 170±2 | 172±2 | NS | *p* < 0.05 | NS |
| Weight (kg) | 72±5 | 71±2 | 72±3 | 70±2 | 81±2 | 68±3 | NS | *p* < 0.05 | NS |
| Body Fat (%) | 34±2 | 17±2 | 33±3 | 17±2 | 36±2 | 23±2 | NS | *p* < 0.05 | NS |
| Total Bone Mineral  Density (g/cm2) | 1.18±0.01 | 1.23±0.02 | 1.21±0.03 | 1.20±0.02 | 1.20±0.02 | 1.17±0.03 | NS | NS | NS |
| DEXA Z-score | 0.15±0.18† | 0.61±0.30 | 0.99±0.29 | 0.30±0.20† | 1.28±0.19 | 0.50±0.32 | NS | NS | *p* < 0.05 |

*Note*: *p* Values refer to significant main effects (*p* < 0.05) identified by two-way (age by activity group) analysis of variance. NS = non-significant (*p* >

0.05). Data are presented as mean±*SE*. †Significantly different from SED 65-86 y group (*p* < 0.05). DEXA = dual-energy x-ray absorptiometry.

Supplementary Table 2. Results of the exercise-stimulated plasma cytokine and chemokine screen in young individuals

**Sedentary Active**

**Analyte Basal (pg/ml) Post-exercise (pg/ml) Basal (pg/ml) Post-exercise (pg/ml) Group effect Exercise effect Interaction**

EGF 36.58±16.06 38.83±14.98 37.87±7.08 38.06±6.41 NS NS NS FGF-2 45.51±7.21 51.60±6.00 55.63±9.51 55.91±5.92 NS NS NS Eotaxin 61.44±6.86 59.31±4.96 63.41±4.34 63.14±3.15 NS NS NS TGF-α ND ND N/A N/A N/A G-CSF 54.78±16.09 58.96±13.36 51.88±8.32 51.01±6.87 NS NS NS Flt-3L 54.50±29.08 53.44±22.18 32.38±10.72 42.33±11.46 NS NS NS GM-CSF 2.31±0.66 3.51±0.78 4.01±1.01 6.00±1.16 NS ***P* < 0.05** NS Fractalkine 63.13±16.11 82.02±15.29 82.74±16.32 93.22±13.90 NS ***P* < 0.05** NS IFNα2 26.37±11.26 30.09±10.36 22.55±4.23 27.21±4.50 NS NS NS IFNγ 11.14±4.79 11.76±4.02 9.16±1.74 12.08±1.82 NS NS NS GRO 190.54±31.28 254.75±39.52 189.15±24.76 315.67±40.10 NS NS NS

IL-10 10.49±2.12 13.62±2.39 12.60±1.40 18.90±2.65 **a,b,c** NS ***P* < 0.05 *P* < 0.05**

MCP-3 14.67±3.53 15.26±3.27 17.55±3.51 19.31±3.09 NS NS NS

IL-12p40 27.93±10.97 25.50±9.36 35.30±16.53 **b** 41.94±17.80 **a,b** NS NS ***P* < 0.05**

MDC 852.76±90.95 797.59±80.62 655.32±46.17 **a,b** 726.72±71.05 **a** NS NS ***P* < 0.05**

IL-12p70 9.09±4.48 8.59±2.63 11.86±3.96 11.36±2.69 NS NS NS IL-13 9.47±5.08 6.67±2.18 5.59±1.13 6.86±1.38 NS NS NS

IL-15 4.13±1.41 4.00±1.35 4.14±1.03 5.57±1.18 **a,b,c** NS ***P* < 0.05 *P* < 0.05**

sCD40L 248.26±20.19 42.53±22.83 280.27±46.57 251.68±17.30 NS NS NS IL-17 11.04±3.98 11.63±3.69 9.31±1.77 9.22±1.59 NS NS NS IL-1RA 43.83±15.24 48.22±14.73 55.02±12.77 59.39±13.95 NS NS NS sIL-2Rα ND ND N/A N/A N/A IL-1α ND 20.10±7.21 23.30±8.14 N/A *P* = 0.062$ N/A IL-9 4.52±1.33 3.64±1.37 3.98±1.30 4.08±1.47 NS NS NS IL-1β 1.19±0.33 1.17±0.20 1.53±0.50 1.80±0.50 NS NS NS IL-2 5.55±1.99 6.63±1.95 12.70±5.20 17.00±6.54 NS ***P* < 0.05** *P* = 0.091

IL-3 0.26±0.07 0.30±0.05 0.20±0.070 0.34±0.07 NS ***P* < 0.05** NS IL-4 12.75±4.84 15.59±3.51 24.84±7.37 30.93±7.58 NS ***P* < 0.05** NS IL-5 0.49±0.14 0.76±0.15 0.63±0.21 0.96±0.20 NS ***P* < 0.05** NS IL-6 2.04±0.34 3.26±0.43 1.61±0.29 3.04±0.35 NS ***P* < 0.05** NS IL-7 2.12±0.53 3.11±0.61 2.99±1.03 3.60±0.82 NS ***P* < 0.05** NS IL-8 3.21±0.54 3.51±0.48 3.24±0.40 3.66±0.45 NS *P* = 0.075 NS IP-10 425.02±30.60 381.24±27.55 411.45±46.90 358.32±47.99 NS ***P* < 0.05** NS

MCP-1 238.83±25.14 234.84±25.86 233.86±19.95 255.12±23.73 NS NS *P* = 0.089

MIP-1α 19.28±6.00 16.60±4.96 7.22±0.97 **a,b** 7.09±0.97 **a,b *P* < 0.05 *P* < 0.05 *P* < 0.05**

MIP-1β 33.31±6.56 34.72±6.15 33.25±5.14 33.82±3.90 NS NS NS TNFα 5.79±1.09 6.18±0.81 5.63±0.40 6.13±0.38 NS ***P* < 0.05** NS TNFβ ND 14.53±5.00 18.15±5.65 N/A ***P* < 0.05$** N/A VEGF 154.69±38.32 159.50±31.97 147.90±26.25 147.34±23.02 NS NS NS PDGF-AA 127.41±21.45 224.91±40.40 250.50±106.71 325.28±40.33 NS NS NS PDGF-BB 1334.30±516.52 2620.75±670.60 2721.42±1533.11 3276.86±634.20 NS NS NS RANTES 4575.35±1083.72 10137.49±2034.04 5237.39±1133.96 18336.68±3000.79 **a,b,c *P* < 0.05 *P* < 0.05 *P* < 0.05**

The indicated significant differences for each effect are shown in bold resulting from a 2-way repeated measures analysis of variance (ANOVA). $*P*-value reflects comparison to ACT basal condition only using a paired t-test. The respective superscript letter indicates a significant interaction effect amongst the labeled samples: versus aSED basal, bSED post-exercise, cACT basal or dACT post-exercise. Data are mean±SEM. N/A, not applicable. ND, not detectable. NS, non-significant.
